# A novel meningioma with tyrosine‐rich crystals in a 6‐year‐old Great Dane

**DOI:** 10.1111/jvim.16789

**Published:** 2023-06-13

**Authors:** Kara Majors, Savannah M. Rocha, Rebecca Windsor, Ronald B. Tjalkens, Julia Engelien, Tawfik Aboellail

**Affiliations:** ^1^ Wheat Ridge Animal Hospital Wheat Ridge Colorado USA; ^2^ Department of Environmental and Radiological Health Sciences Colorado State University Fort Collins Colorado USA; ^3^ Department of Microbiology, Immunology, and Pathology Colorado State University Fort Collins Colorado USA

**Keywords:** atypical, brain tumor, crystal, dog, frontal, neoplasia, olfactory

## Abstract

A 6‐year‐old female spayed Great Dane was evaluated for acute onset cluster seizures. Magnetic resonance imaging (MRI) identified a mass in the olfactory bulbs with a large mucoid component caudal to the primary mass. The mass was removed via transfrontal craniotomy and histopathology revealed a tyrosine crystalline‐rich, fibrous meningioma with a high mitotic index. Repeat MRI at 6 months showed no detectable tumor regrowth. The dog is clinically normal with no seizures at the time of publication 10 months after surgery. This meningioma subtype is rare in humans. This unique meningioma occurred in a dog of younger age and uncommon breed for intracranial meningioma. Biological progression of this tumor subtype is unknown; however, growth rate might be slow despite the high mitotic index.

**Abbreviations**
CCD: charge‐coupled device
DAPI: 4′,6‐diamino‐2‐phenylindole
ER2: Epitope Retrieval Buffer 2
FLAIR: fluid‐attenuated inversion recovery
GRE: gradient echo
MRI: magnetic resonance imaging
PBS: phosphate‐buffered saline
PMMA: polymethylmethacrylate
T1W: T1‐weighted
T2W: T2‐weighted
TH: tyrosine hydroxylase
WHO: World Health Organization

## 1 INTRODUCTION

Intracranial neoplasia occurs in 2.8% to 4% of dogs, and meningiomas comprise ~50% of brain tumors in dogs.^1^, ^2^, ^3^, ^4^, ^5^, ^6^, ^7^ The most common clinical signs associated with intracranial neoplasia in dogs include seizures, behavior changes, visual deficits and motor deficits depending on the location of the tumor.^1^, ^2^, ^6^, ^7^, ^8^, ^9^ The majority of meningiomas in dogs occur in the forebrain, and 70% of forebrain tumors originate in the olfactory bulbs or frontal lobes.^3^, ^5^, ^6^, ^7^, ^8^, ^9^ Meningiomas occur most commonly in large breed dogs (Golden Retrievers, Labrador Retrievers, Boxers, and German Shepherd Dogs) with a median age of onset of 11 years.^3^, ^5^, ^6^, ^7^, ^8^, ^9^, ^10^ Meningothelial meningiomas are the most common subtype in dogs; other subtypes with similarly benign biological behavior include fibroblastic, psammomatous, microcystic, angiomatous, and transitional meningiomas.^2^, ^3^, ^4^, ^9^ Anaplastic or atypical meningiomas are generally considered more biologically aggressive.^2^, ^3^, ^4^, ^9^ Treatment options include surgery and radiation therapy with survival times ranging from 5 months to more than 3 years with surgical resection alone and 9‐12 months with radiation therapy.^8^, ^9^, ^10^, ^11^, ^12^, ^13^, ^14^, ^15^ Survival time might be increased by combining surgical resection with postoperative radiation therapy.^11^ The purpose of this report was to describe a unique meningioma in a dog of younger age and atypical breed. This tumor is suspected to have benign biological behavior despite exhibiting a high mitotic index.

## 2 CASE DESCRIPTION

### 2.1 Clinical presentation

A 6‐year‐old female spayed Great Dane was presented to a private specialty hospital for acute onset of 3 generalized tonic‐clonic seizures that occurred within the 24‐hour period before presentation. There was no history of seizures, abnormal medical history, or potential toxin exposure. In retrospect the owners noted some mild lethargy in the weeks to months before the seizures began.

On physical examination, the dog was tachycardic (140 beats/min) and mildly tachypneic (40 breaths/min). The rectal temperature was normal. Abnormalities were not detected on general physical examination. After initial recovery from the seizures, the dog had a normal neurological examination other than mild cervical hyperesthesia.

### 2.2 Diagnostic findings

Complete blood count (CBC) revealed a mild eosinophilia (1125/μL; reference interval [RI], 0‐990/μL). Serum biochemistry revealed an elevated alanine aminotransferase (120 U/L; RI, 17‐115 U/L), elevated aspartate transaminase (47 U/L; RI, 11‐46 U/L), and elevated alkaline phosphatase (752 U/L; RI, 8‐196 U/L). Urinalysis, total T4, and ELISA 4Dx snap test (*Anaplasma phagocytophilum*, *Anaplasma platys*, *Erlichia canis*, *Erlichia ewingii*, *Borrelia burgdorferi*, and *Dirofilaria immitis*) were normal. Thoracic radiographs showed normal cardiothoracic structures and incidental bilateral osteophytosis of caudal glenoid cavities and humeral heads.

**FIGURE 1:**
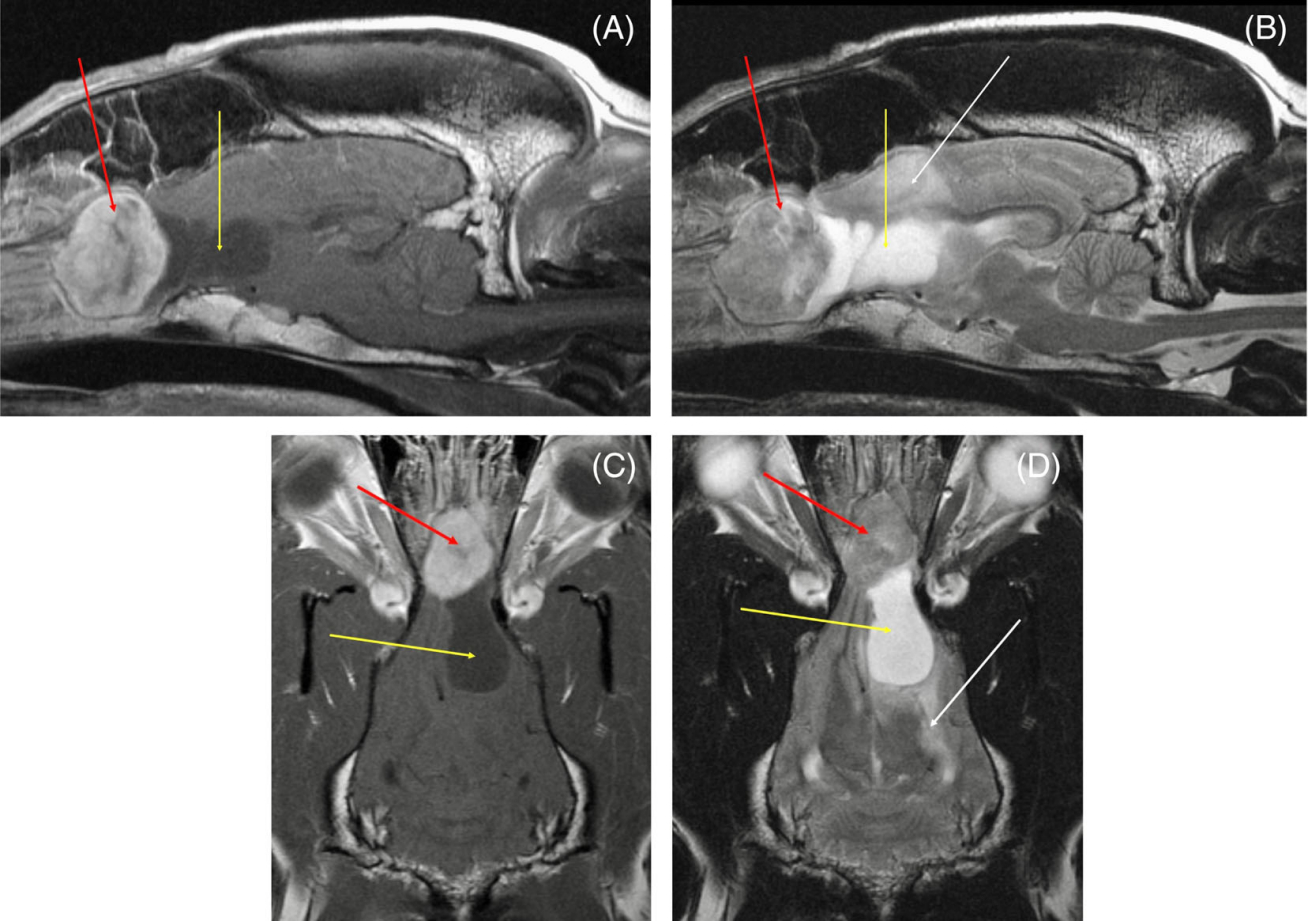
T1W postcontrast sagittal image (A) showing the fibrous portion of the mass (red arrow) and mucoid component of the mass (yellow arrow). T2W sagittal image (B) showing the fibrous portion of the mass (red arrow), mucoid component of the mass (yellow arrow), and peritumoral edema (white arrow). T1W postcontrast dorsal plane image (C) showing the fibrous portion of the mass (red arrow) and mucoid component of the mass (yellow arrow). T2W dorsal plane image (D) showing the fibrous portion of the mass (red arrow), mucoid component of the mass (yellow arrow), and peritumoral edema (white arrow).

Magnetic resonance imaging (MRI) of the brain (1.5 Tesla Siemens MAGNETOM Symphony) was acquired using T2‐weighted (T2W) sagittal, transverse, and dorsal planes; T1‐weighted (T1W) pre‐ and postcontrast transverse and sagittal planes and T1W postcontrast dorsal plane; and fluid‐attenuated inversion recovery (FLAIR) and gradient echo (GRE) in a transverse plane. MRI revealed a large olfactory bulb/frontal lobe mass with two distinct parts (Figure 1). The rostral portion contained a T2W iso/hyperintense, FLAIR iso/hyperintense, T1W precontrast isointense, strongly and uniformly T1W postcontrast enhancing mass in the olfactory bulbs (larger on the right side) measuring 2.7 cm rostrocaudal × 3 cm dorsoventral × 2 cm mediolateral. The second portion of the mass extended into the left frontal lobe, measuring 3.2 cm rostrocaudal × 1.6 cm dorsoventral × 1.7 cm mediolateral. This portion of the mass was markedly T2W hyperintense, T1W and FLAIR hypointense, and noncontrast enhancing on T1W postcontrast, consistent with liquid or mucoid/gelatinous material. Marked peritumoral T2W and FLAIR hyperintensity extended diffusely throughout the left cerebral hemisphere following predominantly the white matter caudally to the level of the occipital cortex consistent with marked peritumoral edema. Mild caudal transtentorial herniation was present.

### 2.3 Treatment

All gross tumor was removed via transfrontal craniotomy. The rostral portion of the mass was firm and well‐defined and contained strand‐like fibrous material with a consistency unlike other meningiomas in dogs based on the experience of the surgeon. The caudal mucoid component was removed via suction and no visible tumor remained. Samples of the mass were submitted for histopathology (Colorado State University Veterinary Diagnostic Laboratory). A polymethylmethacrylate (PMMA) skull cap (Surgical Simplex P radiopaque bone cement, Stryker) was placed over the craniectomy defect before closure. There were no anesthetic complications, and the dog was discharged 48 hours after surgery with instructions for phenobarbital (1.8 mg/kg PO every 12 hours), gabapentin (5.4 mg/kg PO every 8 hours), cefpodoxime (7.2 mg/kg PO every 24 hours), and prednisone (0.35 mg/kg PO every 12 hours).

### 2.4 Histopathology

**FIGURE 2:**
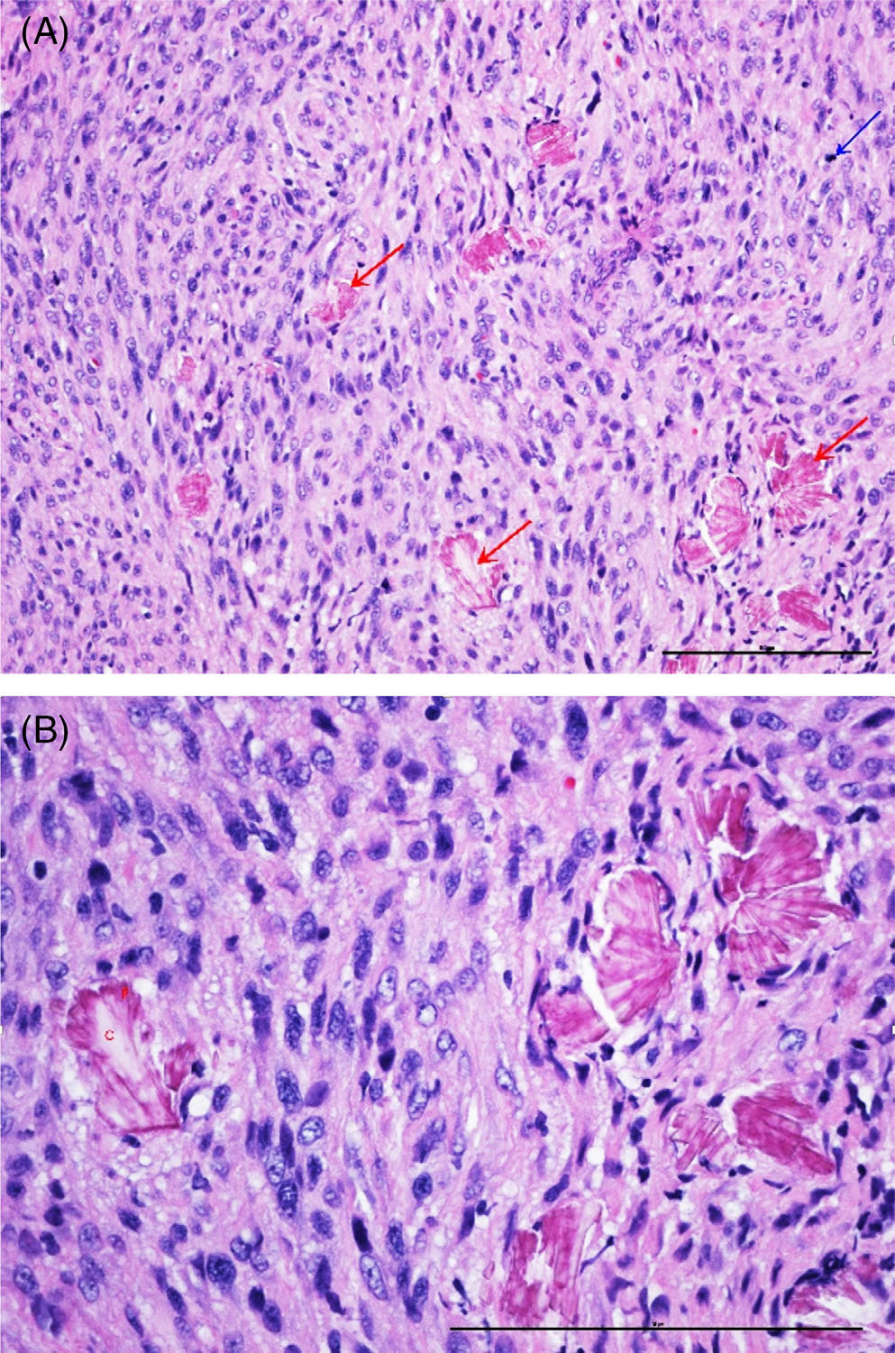
(A) Photomicrograph of the forebrain mass. The neoplasm is highly cellular and is composed of interlacing bundles of plump spindle cells with mitotic activity (blue arrow), and embedded multifocal tyrosine crystals (red arrows). Scale = 100 μm. (B) Higher magnification of the mass demonstrates multifocal extracellular crystalloid material exhibiting floret‐like arrangement with many 2 to 3 μm petals (P) that surround 30 μm elongated central core (C). Scale = 100 μm.

Histologically, the neoplasm exhibited a Schwannian arrangement where spindle cells were arranged in fascicular architecture and occasionally showed palisading of their nuclei (Figure 2). Individual tumor cells had variably distinct cell margins and scant to moderate amounts of eosinophilic cytoplasm. Most of the neoplastic nuclei were round to oval with multifocal nuclear atypia. There were occasional karyomegalic profiles and rare binucleate to multinucleate giant cells. Mitotic index was high and ranged between 28 and 30 mitotic figures per a unit area of 2.37 mm^2^ (equivalent to 10 high‐power microscopic fields). There was abundant deposition of extracellular and intracellular eosinophilic crystalloid material multifocally throughout the tumor, more numerous in some areas than others. These crystalloid deposits were primarily arranged in parallel with the fascicles in floret‐like arrangements with numerous petals that surrounded an elongated central core. The crystal aggregates varied widely in size with individual petals measuring 2 to 3 μm in size and large conglomerates measuring up to 30 μm in width. These crystals were strongly reminiscent of tyrosine‐like crystals, seen most commonly in salivary gland neoplasia. Many neoplastic cells contained small hypereosinophilic crystalline material.

### 2.5 Immunofluorescence identification of tyrosine hydroxylase crystals

Paraffin‐embedded canine brain tissue was sectioned at 5‐μm thickness and mounted onto polyionic slides. Slides were deparaffinized and tissue sections were immunofluorescently labeled on a fully automated Leica Bond RX_m_ robotic staining system. Epitope retrieval was performed through application of Bond Epitope Retrieval Buffer 2 (ER2) for 20 minutes in conjunction with heat. Sections were then incubated with tyrosine hydroxylase (TH; 1:500) diluted in 0.1% Triton‐X containing phosphate‐buffered saline (PBS). Sections were stained for 4′,6‐diamidino‐2‐phenylindole (DAPI; Sigma) and mounted on glass coverslips with Prolong Gold Anti‐fade mounting media. Mounting media was allowed to cure at room temperature in the dark for 24 hours. Sections were stored at 4°C until imaged.

Full section montage images were acquired on a fully‐automated motorized stage VS200 Olympus microscope equipped with a Hamamatsu ORCA‐Fusion camera and collected using Olympus CellSens software. Whole tissue section montages were generated by compiling ×200 images acquired by using an Olympus UPLX‐Apochomat ×20 (0.8 N.A.) air objective. High magnification inset images were acquired using an Olympus UPLX‐Apochromat ×60 oil immersion objective (1.42 N.A.) z‐stack with post acquisition deconvolution through Weiner method provided on the CellSens platform. All images were acquired simultaneously by a single investigator where charge‐coupled device (CCD) imaging parameters (binning, exposure, and gain) were held consistent to reduce variability in detected fluorescence intensity.

**FIGURE 3:**
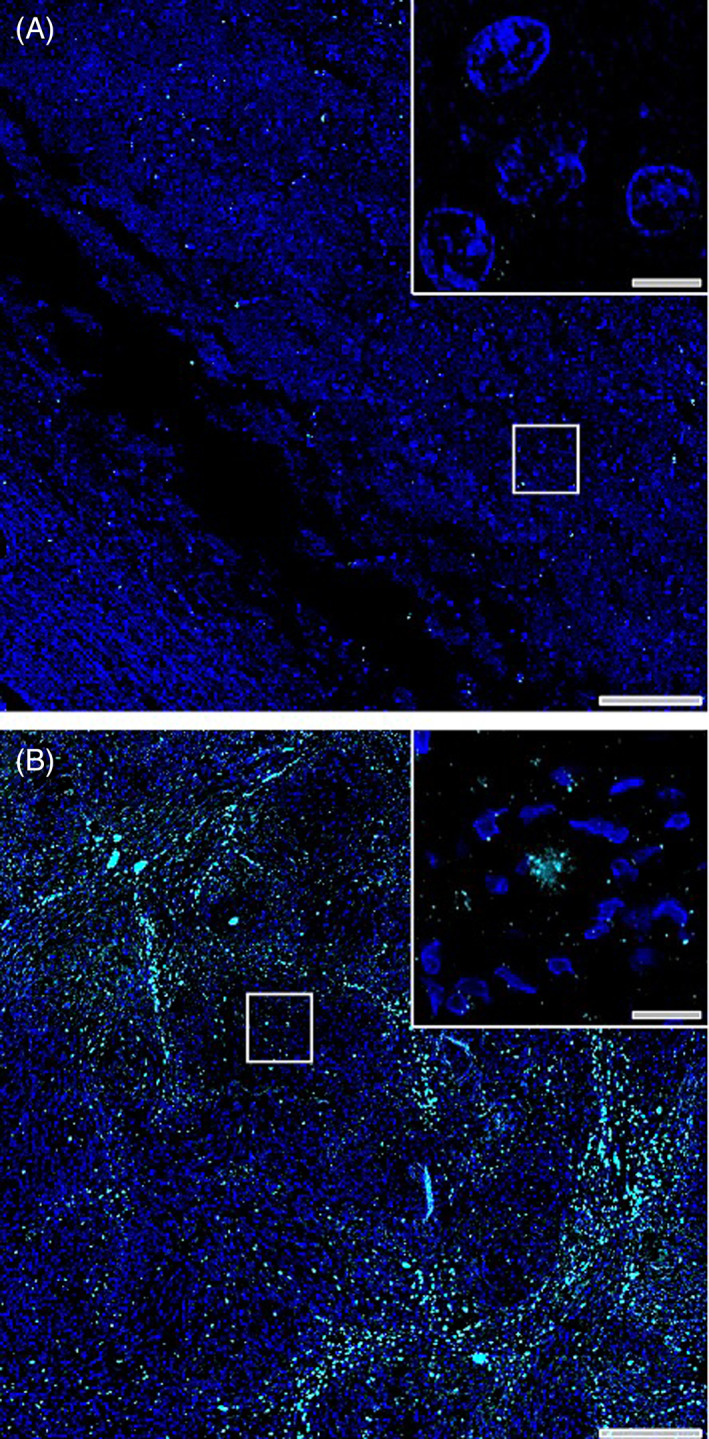
Immunofluorescence staining of tyrosine hydroxylase‐rich crystals. Immunofluorescence staining of tyrosine hydroxylase (cyan) and cellular nuclei (DAPI, blue) in tissue with unremarkable brain pathology (A) and within biopsy tissue section of case study patient (B). Scale bars in low magnification montage images = 200 μM and scale bars in high magnification insets = 10 μM.

Immunofluorescence staining for TH within tissue sections of an animal with unremarkable brain pathology (Figure 3A) show minimal TH^+^ puncta, where positive staining is indicative of non‐nucleated red blood cells bordered by epithelial cells, apparent by the elongated DAPI^+^ nuclei. TH staining within animal tissue sections revealed multifocal extracellular tyrosine‐rich crystalline structures that were apparent in multiple ×60 high magnification imaging planes, confirming crystalline‐like diffraction patterns of intensity, that were not bordered by epithelial cells (Figure 3B).

### 2.6 Outcome

**FIGURE 4:**
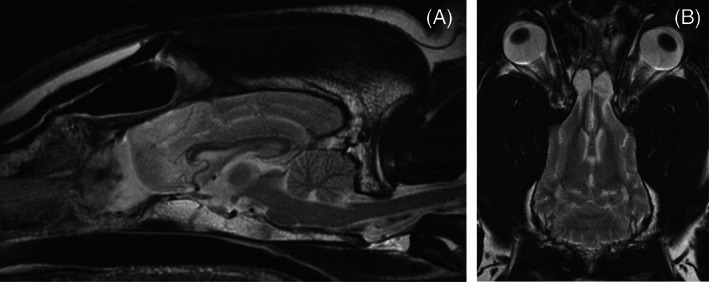
Six month postoperative sagittal (A) and dorsal (B) T2‐weighted MRI images.

Administration of prednisone was gradually reduced and discontinued by 3 weeks after surgery and phenobarbital (1.8 mg/kg PO every 12 hours) administration was continued long‐term. Brain MRI was repeated 6 months after craniotomy (Figure 4). There was no detectable tumor regrowth. An area of bilaterally symmetrical T2W hyperintensity was identified in the rostral olfactory bulbs which was expected to be consistent with normal postoperative change but could represent early recurrence of the mucoid component of the mass. The dog is clinically normal with no seizures at the time of manuscript submission (10 months postcraniotomy).

## 3 DISCUSSION

This case report describes a meningioma subtype exhibiting tyrosine rich crystals. Only 3 tumors of this subtype are reported in humans, including 2 intracranial and 1 spinal tyrosine‐rich meningioma.^16^, ^17^, ^18^ Clinical signs were apparent for 6 months to 2 years in these humans before diagnosis. This subtype exhibits magenta‐colored extracellular deposits that appear “petal‐shaped” on electron microscopy. These eosinophilic crystals have blunt ends that are radially arranged and are presumed to be tyrosine‐rich crystals based on their appearance and positivity with Millon staining, which indicates the presence of tyrosine residues.^16^, ^17^ In 1 patient, the crystalline material was unevenly distributed with more numerous depositions occurring in areas with degenerative changes characterized by fibrosis, myxoid stromal change, and increased chronic inflammatory infiltrates.^18^ Clinical outcome and long‐term follow‐up of the 3 humans with tyrosine‐rich meningiomas was not reported.^16^, ^17^, ^18^ The presence of tyrosine‐like crystalloid material in meningiomas does not yet have prognostic relevance in humans because of their scarcity in the literature.

Canine and human meningiomas share many histologic and imaging similarities, and previous studies have attempted to apply WHO classification of meningiomas to dogs.^2^, ^3^, ^4^, ^9^ No correlation between histologic grade and prognosis has been identified.^4^ Evaluation of immunohistochemical staining patterns to better classify meningiomas has been performed; however, further studies are needed to evaluate clinical relevance.^19^

Treatment of intracranial meningiomas can consist of surgical resection, radiation, and less commonly administration of chemotherapies such as hydroxyurea.^7^, ^8^, ^9^, ^10^, ^11^, ^12^, ^13^, ^14^, ^15^, ^20^ There are conflicting reports regarding survival time with some studies stating no significant difference in outcome between dogs with surgical resection alone compared with surgery with chemotherapy, radiation, or a combination of these therapies and another showing prolonged survival time with postoperative radiation.^9^, ^11^, ^12^, ^13^, ^14^, ^15^ One study examined the influence of tumor cell proliferation receptors on effectiveness of radiation therapy and found that meningiomas with reduced tumor proliferation index were 9 times more likely to be controlled by radiation, which might explain improved survival times with radiation for some meningiomas.^21^ The dog in this case report received surgical resection alone and will be monitored with recheck examinations and repeat MRIs as clinically indicated. This dog was younger than the median age for dogs with meningiomas and in a breed that has not been commonly reported,^5^, ^6^, ^7^, ^8^, ^9^ and it is possible that this tumor has a different biological pattern than other canine meningiomas. It is also important to note that despite the high mitotic index observed on histopathology, there are no neurological signs or MRI evidence of regrowth in the follow‐up period to date.

## CONFLICT OF INTEREST DECLARATION

Authors declare no conflict of interest.

## OFF‐LABEL ANTIMICROBIAL DECLARATION

Authors declare no off‐label use of antimicrobials.

## INSTITUTIONAL ANIMAL CARE AND USE COMMITTEE (IACUC) OR OTHER APPROVAL DECLARATION

Authors declare no IACUC or other approval was needed.

## HUMAN ETHICS APPROVAL DECLARATION

Authors declare human ethics approval was not needed for this study.
